# Avaliação do Seguimento de um Ano dos Pacientes Incluídos no Registro Brasileiro de Síndromes Coronárias Agudas (ACCEPT)

**DOI:** 10.36660/abc.20190879

**Published:** 2020-06-29

**Authors:** Pedro Gabriel Melo de Barros e Silva, Otavio Berwanger, Elizabete Silva dos Santos, Antônio Carlos Sobral Sousa, Margaret Assad Cavalcante, Pedro Beraldo de Andrade, Fernando Carvalho Neuenschwander, Hugo Vargas, Jorge Ilha Guimarães, Jadelson Andrade, Angelo Amato Vincenzo de Paola, Marcus Vinicius Bolivar Malachias, Luiz Alberto Piva e Mattos, Dalton Bertolim Precoma, Fernando Bacal, Oscar Pereira Dutra

**Affiliations:** 1 Hospital do Coração São PauloSP Brasil Hospital do Coração - Pesquisa Clínica, São Paulo, SP - Brasil; 2 Hospital Samaritano Paulista São PauloSP Brasil Hospital Samaritano Paulista – Cardiologia, São Paulo, SP - Brasil; 3 Hospital Israelita Albert Einstein São PauloSP Brasil Hospital Israelita Albert Einstein, São Paulo, SP - Brasil; 4 Instituto Dante Pazzanese de Cardiologia São PauloSP Brasil Instituto Dante Pazzanese de Cardiologia,São Paulo, SP - Brasil; 5 PPGCS HU Universidade Federal de Sergipe AracajuSE Brasil PPGCS e HU da Universidade Federal de Sergipe (UFS), Aracaju, SE - Brasil; 6 Hospital São Lucas Rede D`Or AracajuSE Brasil Hospital São Lucas - Rede D`Or, Aracaju, SE - Brasil; 7 Universidade do Oeste Paulista Presidente PrudenteSP Brasil Universidade do Oeste Paulista (Unoeste), Presidente Prudente, SP - Brasil; 8 Hospital Regional de Presidente Prudente Presidente PrudenteSP Brasil Hospital Regional de Presidente Prudente, Presidente Prudente, SP - Brasil; 9 Faculdade de Medicina de Marilia MariliaSP Brasil Faculdade de Medicina de Marilia, Marilia, SP - Brasil; 10 Santa Casa de Marília MaríliaSP Brasil Santa Casa de Marília, Marília, SP - Brasil; 11 Hospital Vera Cruz Belo HorizonteMG Brasil Hospital Vera Cruz, Belo Horizonte, MG - Brasil; 12 Hospital São Vicente de Paulo Passo FundoRS Brasil Hospital São Vicente de Paulo, Passo Fundo, RS - Brasil; 13 Sociedade Brasileira de Cardiologia Rio de JaneiroRJ Brasil Sociedade Brasileira de Cardiologia, Rio de Janeiro, RJ - Brasil; 14 Hospital da Bahia SalvadorBA Brasil Hospital da Bahia, Salvador, BA - Brasil; 15 Universidade Federal de São Paulo Escola Paulista de Medicina São PauloSP Brasil Universidade Federal de São Paulo Escola Paulista de Medicina, São Paulo, SP - Brasil; 16 Faculdade de Ciências Médicas de Minas Gerais Belo HorizonteMG Brasil Faculdade de Ciências Médicas de Minas Gerais, Belo Horizonte, MG - Brasil; 17 Instituto de Hipertensão Arterial Belo HorizonteMG Brasil Instituto de Hipertensão Arterial - Diretoria Clínica,Belo Horizonte, MG - Brasil; 18 Rede D`Or São PauloSP Brasil Rede D`Or, São Paulo, SP – Brasil; 19 Rede D`Or RecifePE Brasil Rede D`Or, Recife, PE - Brasil; 20 Pontifícia Universidade Católica do Paraná Escola de Medicina CuritibaPR Brasil Pontifícia Universidade Católica do Paraná - Escola de Medicina, Curitiba, PR – Brasil; 21 Sociedade Hospitalar Angelina Caron Campina Grande do SulPR Brasil Sociedade Hospitalar Angelina Caron – Cardiologia, Campina Grande do Sul, PR - Brasil; 22 Universidade de São Paulo Faculdade de Medicina Hospital das Clínicas São PauloSP Brasil Universidade de São Paulo Faculdade de Medicina Hospital das Clínicas Instituto do Coração, São Paulo, SP - Brasil; 23 Instituto de Cardiologia Fundação Universitária de Cardiologia do Rio Grande do Sul Porto AlegreRS Brasil Instituto de Cardiologia – Fundação Universitária de Cardiologia do Rio Grande do Sul,Porto Alegre, RS – Brasil

**Keywords:** Síndrome Coronariana Aguda, Infarto do Miocárdio, Fatores de Risco, Estudos Multicêntricos, Registros Médicos/estatística &dados numéricos.

## Abstract

**Fundamento:**

Existe carência de informações prospectivas sobre a evolução em um ano após uma síndrome coronária aguda (SCA) em uma grande amostra de pacientes brasileiros.

**Objetivos:**

Avaliar a prescrição de terapias baseadas em evidência, a ocorrência de desfechos graves e os preditores para estes desfechos em um registro brasileiro multicêntrico de pacientes com SCA.

**Métodos:**

O ACCEPT é um estudo observacional prospectivo que incluiu pacientes internados com diagnóstico de SCA em 47 hospitais brasileiros. Os pacientes foram seguidos por 1 ano e coletou-se dados sobre prescrição médica e ocorrência de eventos cardiovasculares maiores (mortalidade cardiovascular, reinfarto e acidente vascular encefálico - AVE). Valores de p < 0,05 foram considerados estatisticamente significantes.

**Resultados:**

Um total de 5.047 pacientes foram incluídos neste registro, de agosto de 2010 até abril de 2014. Foi confirmado o diagnóstico de SCA em 4.782 pacientes (94,7%) e, dentre os 3 diagnósticos possíveis, o mais comum foi SCA com elevação do segmento ST (35,8%). A taxa de eventos cardiovasculares maiores foi de 13,6 % em 1 ano. A prescrição completa de terapias baseadas em evidência na admissão hospitalar foi de 62,1%. Idade, atendimento público, infarto agudo do miocárdio, AVE, insuficiência renal, diabetes e qualidade da terapia estiveram associados de forma independente à ocorrência de eventos cardiovasculares maiores.

**Conclusões:**

No seguimento de 1 ano do registro ACCEPT, mais de 10% dos pacientes apresentaram eventos cardiovasculares maiores e esta taxa variou de acordo com a qualidade da terapia. Há necessidade da elaboração de estratégias para melhorar o uso de terapias baseadas em evidência no sentido de minimizar os eventos cardiovasculares na população brasileira. (Arq Bras Cardiol. 2020; 114(6):995-1003)

## Introdução

O grupo das doenças cardiovasculares, particularmente a síndrome coronária aguda (SCA), representa a principal causa de mortalidade e incapacidade no Brasil e no mundo.^1 - 3^ Além da elevada frequência atual, há perspectiva de crescimento deste grupo de doenças em países em desenvolvimento como o Brasil. ^1 - 5^ Apesar da elevada morbimortalidade da SCA no cenário atual, diversas estratégias de comprovada eficácia para reduzir o risco de complicações nestes pacientes têm sido desenvolvidas.^6 , 7^ Entretanto, há falhas na aplicação das terapias baseadas em evidência em pacientes com SCA, conforme identificado em registros prévios de prática clínica.^8 - 10^ Tais registros multicêntricos avaliaram predominantemente o período intra-hospitalar ou decorridos 30 dias do evento agudo registrando-se, todavia, a carência de informações a longo prazo sobre o acompanhamento destes pacientes^8 - 10^ Dentre estes registros prévios com seguimento de até 30 dias, há a publicação de dados parciais (sem amostra completa) do estudo ACCEPT.^10^ Conforme previamente reportado na publicação dos dados parciais de 30 dias.^10^ o ACCEPT tinha como plano continuar o estudo, arrolando um número maior de pacientes e incluir dados do seguimento de 12 meses. Dessa forma, a presente análise realizou, novamente, a avaliação das características basais e a adesão inicial da prescrição médica a terapias baseadas em evidências numa população maior (aproximadamente o dobro de pacientes em relação à publicação inicial com dados intermediários) e incluiu dados sobre a taxa de ocorrência de desfechos clínicos graves durante o seguimento.

## Objetivos

Além dos resultados finais em 30 dias com a população completa do estudo, esta análise do seguimento de 1 ano tem os seguintes objetivos específicos:

- Avaliar a taxa de eventos cardiovasculares maiores em 12 meses numa população de pacientes brasileiros pós-SCA;

- Avaliar a taxa de conformidade da prescrição médica a terapias baseadas em evidência em 12 meses numa população de pacientes brasileiros pós-SCA;

- Identificar preditores de eventos cardiovasculares maiores em 12 meses numa população de pacientes brasileiros pós-SCA.

## Métodos

### Delineamento do estudo

O Registro ACCEPT (Acute Coronary Care Evaluation of Practice Registry) é um projeto idealizado pela Sociedade Brasileira de Cardiologia (SBC), cujos métodos foram previamente publicados.^10 , 11^ De forma resumida, trata-se de pesquisa prospectiva, voluntária, multicêntrica que reuniu 53 centros das 5 regiões brasileiras com a seguinte distribuição: sudeste (50,9%), nordeste (13,2%), sul (24,5%), centro oeste (5,7%) e norte (5,7%). A inclusão de pacientes ocorreu de agosto de 2010 até abril de 2014, em centros hospitalares com assistência pública (Sistema Único de Saúde - SUS), de saúde suplementar (operadoras de saúde) ou privados, de acordo com a seguinte distribuição: SUS 2669/4782 (55,8%), saúde suplementar 1968/4782 (41,2%) e privado 145/4782 (3%).

### Participantes do estudo

Foram incluídos pacientes na vigência do diagnóstico de SCA nas suas diferentes formas de apresentação: angina instável (AI), infarto agudo do miocárdio sem supradesnível do segmento ST (IAM sSST) e com supradesnível do segmento ST (IAM cSST). Os principais critérios de inclusão foram sintomas isquêmicos suspeitos de SCA associados a alterações no eletrocardiograma (ECG) compatíveis com alteração isquêmica e/ou marcadores de lesão miocárdica, acima do limite superior da normalidade. Foram excluídos pacientes transferidos de outras instituições com mais de 12 horas do início dos sintomas.

### Procedimentos do estudo e variáveis analisadas

Os procedimentos do estudo e variáveis analisadas no estudo ACCEPT foram previamente publicados.^10 , 11^ De forma sucinta, a coleta de dados ocorreu na internação, com dados da admissão (visita índice), e também foi feita uma segunda coleta de dados de 7 dias ou até a alta hospitalar (o que ocorresse primeiro). Após estas duas primeiras visitas, o estudo planejou visitas de 30 dias, 6 meses e 12 meses, que poderiam ocorrer de forma presencial na rotina assistencial ou por telefone.

Tendo a característica de um estudo pragmático, a identificação de comorbidade dos pacientes (ex.: hipertensão arterial, dislipidemia) poderia ser realizada da seguinte forma: relato pelo paciente, uso de medicamento (anti-hipertensivo, hipolipemiante) ou avaliação do investigador (neste último, os centros foram orientados a seguirem as recomendações de critérios diagnósticos adotadas pelas diretrizes vigentes da Sociedade Brasileira de Cardiologia). Características do exame físico poderiam ser obtidas por mensuração direta (a obesidade foi definida por IMC > 30 Kg/m^2^). Demais critérios se basearam no registro em prontuário de uma variável coletada através de questionamento em entrevista (ex.: estresse, ex-tabagista se cessação > 6 meses).

O esquema terapêutico baseado em evidência que foi considerado no ACCEPT não se modificou durante o estudo e se baseou em diretrizes vigentes.^6 , 7^ Este esquema terapêutico pode ser dividido da seguinte forma:

- Internação do evento índice: Dupla antiagregação, anticoagulante parenteral, estatina e betabloqueador com adição da terapia de reperfusão nos casos de IAM cSST.

- Terapia ambulatorial (pós-alta): Dupla antiagregação, estatina, betabloqueador e IECA/BRA.

Os desfechos cardiovasculares de interesse analisados na população incluída foram: mortalidade cardiovascular, parada cardíaca não fatal, reinfarto e acidente vascular encefálico (AVE).^10 , 11^ Estes desfechos foram reportados pelo investigador de acordo com critérios recomendados,^10 , 11^ sem utilização de um comitê independente para adjudicação de eventos.

### Análise estatística

A avaliação da normalidade de distribuição de variáveis contínuas foi realizada através de histogramas. Variáveis contínuas de distribuição normal foram descritas como média ± desvio padrão. As médias foram comparadas entre os três grupos de diagnósticos utilizando a análise de variância (Anova). Variáveis categóricas foram descritas como frequências absolutas e relativas. Proporções foram comparadas pelo teste de Qui-quadrado ou o Teste (exato) de Fisher-Freeman-Halton. Modelos de Equações de Estimação Generalizada (EEG) foram utilizados para avaliar a terapia medicamentosa ao longo do tempo. Com a finalidade de comparar os eventos cardiovasculares maiores, de acordo com o diagnóstico final, foram utilizados o modelo de riscos proporcionais de Cox e o gráfico de Kaplan-Meier. A identificação de preditores independentes de eventos combinados (AVE, reinfarto e óbito) foi realizada por modelos de risco proporcionais de Cox com diagnóstico final e o fator *baseline* avaliado. Esta análise de preditores foi realizada inicialmente de forma univariada e as variáveis com valor de p <0,15 foram incluídas na análise multivariada. Valores de p apresentados são do tipo bilateral e p < 0,05 foi considerado estatisticamente significante nas análises finais. Adicionalmente, foi realizado teste de interação entre as variáveis selecionadas na análise multivariada. Todas as análises foram realizadas com auxílio do programa R, versão 3.6.1.

## Resultados

Entre agosto de 2010 e abril de 2014, 5.047 pacientes foram recrutados nesse registro nacional, sendo 265 destes (5,25%) portadores de dor torácica a esclarecer e excluídos do seguimento clínico por não preencherem os critérios de inclusão da pesquisa. Sendo assim, 4.782 pacientes portadores de SCA foram efetivamente incluídos na análise e seguidos neste registro prospectivo, em 53 centros hospitalares das 5 regiões brasileiras. Em um total de 410 pacientes (8,6%) não foi possível obter informação final de 12 meses.

### Características basais

O perfil clínico dos pacientes evidenciou a inclusão de aproximadamente 70% dos pacientes com diagnóstico de IAM na admissão, quase um terço de diabéticos, e próximo de 90% com a evidência de ao menos um fator de risco presente, sendo hipertensão arterial sistêmica o mais comum deles ( Tabela 1 ).

**Tabela 1:** – Características basais dos pacientes incluídos de acordo com tipo de síndrome coronária aguda

Diagnóstico Final do Paciente					

	Angina Instável (n=1453)	IAM sem supra de ST (n=1615)	IAM com supra de ST (n=1714)	Total (n=4782)	Valor de p
Idade; média ± DP	63,9 ± 11,9 (n=1449)	64,7 ± 12,4 (n=1603)	60,8 ± 12,4 (n=1702)	63,1 ± 12,4 (n=4754)	<0,001^(1)^
Sexo (Feminino)	588/1453 (40,5%)	489/1615 (30,3%)	460/1714 (26,8%)	1537/4782 (32,1%)	<0,001
Transferido de outro serviço (Sim)	179/1451 (12,3%)	393/1614 (24,3%)	803/1713 (46,9%)	1375/4778 (28,8%)	<0,001
Atendimento (Saúde Suplementar/Particular)	757/1453 (52,1%)	775/1615 (48%)	581/1714 (33,9%)	2113/4782 (44,2%)	<0,001
Pressão Arterial Sistólica; média ± DP	138,1 ± 24,1 (n=1452)	137,9 ± 28 (n=1615)	131,5 ± 26 (n=1713)	135,7 ± 26,4 (n=4780)	<0,001^(1)^
Pressão Arterial Diastólica; média ± DP	81,4 ± 13,9 (n=1452)	81,3 ± 16,4 (n=1615)	80,4 ± 16,4 (n=1713)	81 ± 15,7 (n=4780)	0,142^(1)^
Frequência Cardíaca ; média ± DP	74,6 ± 15,3 (n=1452)	77,6 ± 18 (n=1615)	79,4 ± 17,2 (n=1713)	77,4 ± 17 (n=4780)	<0,001^(1)^
Dislipidemia	971/1453 (66,8%)	915/1615 (56,7%)	734/1713 (42,8%)	2620/4781 (54,8%)	<0,001
IAM prévio	507/1451 (34,9%)	535/1614 (33,1%)	267/1713 (15,6%)	1309/4778 (27,4%)	<0,001
História de Angina	774/1452 (53,3%)	554/1614 (34,3%)	406/1713 (23,7%)	1734/4779 (36,3%)	<0,001
Hipertensão	1197/1453 (82,4%)	1252/1615 (77,5%)	1116/1713 (65,1%)	3565/4781 (74,6%)	<0,001
História familiar de doença coronária	643/1453 (44,3%)	658/1615 (40,7%)	699/1713 (40,8%)	2000/4781 (41,8%)	0,081
AVE	137/1453 (9,4%)	125/1615 (7,7%)	98/1713 (5,7%)	360/4781 (7,5%)	<0,001
Estresse e/ou Depressão	506/1451 (34,9%)	419/1614 (26%)	466/1713 (27,2%)	1391/4778 (29,1%)	<0,001
Insuficiência renal	88/1452 (6,1%)	99/1615 (6,1%)	72/1713 (4,2%)	259/4780 (5,4%)	0,021
Diabetes Mellitus	477/1453 (32,8%)	582/1615 (36%)	453/1713 (26,4%)	1512/4781 (31,6%)	<0,001
Diabetes em uso de insulina	134/474 (28,3%)	150/582 (25,8%)	84/453 (18,5%)	368/1509 (24,4%)	-
Insuficiência cardíaca	180/1452 (12,4%)	156/1615 (9,7%)	87/1713 (5,1%)	423/4780 (8,8%)	<0,001
Intervenção coronária percutânea	489/1450 (33,7%)	406/1614 (25,2%)	209/1713 (12,2%)	1104/4777 (23,1%)	<0,001
Cirurgia de RM	223/1452 (15,4%)	213/1615 (13,2%)	68/1713 (4%)	504/4780 (10,5%)	<0,001
Uso prévio de AAS	861/1453 (59,3%)	703/1615 (43,5%)	383/1713 (22,4%)	1947/4781 (40,7%)	<0,001
Obesidade Abdominal	531/1452 (36,6%)	552/1615 (34,2%)	521/1713 (30,4%)	1604/4780 (33,6%)	0,001
Sedentarismo	949/1453 (65,3%)	968/1615 (59,9%)	962/1713 (56,2%)	2879/4781 (60,2%)	<0,001
Doença arterial periférica	130/1453 (8,9%)	135/1615 (8,4%)	126/1713 (7,4%)	391/4781 (8,2%)	0,252
Tabagismo					
Nunca	761/1453 (52,4%)	756/1615 (46,8%)	664/1713 (38,8%)	2181/4781 (45,6%)	<0,001
Ex-tabagista	487/1453 (33,5%)	503/1615 (31,1%)	387/1713 (22,6%)	1377/4781 (28,8%)	
Atual	205/1453 (14,1%)	356/1615 (22%)	662/1713 (38,6%)	1223/4781 (25,6%)	

*Valor de p: Teste Qui-quadrado. (1) Teste ANOVA.*

### Adesão da prescrição médica a terapias baseadas em evidência

A prescrição adotada logo após a admissão demonstra que a adesão completa aos medicamentos recomendados nas diretrizes vigentes foi de 62,1 % ( tabela 2 ). Esta adesão inclui dupla terapia antiplaquetária (aspirina/inibidor da P2Y12) associada a anticoagulante parenteral, estatina e betabloqueador.

**Tabela 2:** – Utilização de medicamentos na fase de admissão dos pacientes com síndrome coronária aguda

Medicação	Angina Instável	IAM sem supra de ST	IAM com supra de ST	Total	p
AAS	1399/1449 (96,5%)	1580/1615 (97,8%)	1688/1713 (98,5%)	4667/4777 (97,7%)	0,001
Betabloqueador	1144/1449 (79%)	1323/1615 (81,9%)	1352/1713 (78,9%)	3819/4777 (79,9%)	0,052
Inibidor P2Y12	1239/1449 (85,5%)	1483/1615 (91,8%)	1671/1713 (97,5%)	4393/4777 (92%)	<0,001
Clopidogrel	1213/1449 (83,7%)	1401/1615 (86,7%)	1531/1713 (89,4%)	4145/4777 (86,8%)	<0,001
Prasugrel	11/1449 (0,8%)	17/1615 (1,1%)	15/1713 (0,9%)	43/4777 (0,9%)	0,685
Ticagrelor	23/1449 (1,6%)	80/1615 (5%)	149/1713 (8,7%)	252/4777 (5,3%)	<0,001
Anticoagulante Parenteral	1151/1449 (79,4%)	1468/1615 (90,9%)	1500/1713 (87,6%)	4119/4777 (86,2%)	<0,001
Enoxaparina	837/1449 (57,8%)	1039/1615 (64,3%)	1086/1713 (63,4%)	2962/4777 (62%)	<0,001
Fondaparinux	113/1449 (7,8%)	206/1615 (12,8%)	174/1713 (10,2%)	493/4777 (10,3%)	<0,001
Heparina não-fracionada	214/1449 (14,8%)	240/1615 (14,9%)	282/1713 (16,5%)	736/4777 (15,4%)	0,319
Inibidores da GPIIb/IIIa	23/1449 (1,6%)	91/1615 (5,6%)	292/1713 (17%)	406/4777 (8,5%)	<0,001
Abciximab	3/1449 (0,2%)	10/1615 (0,6%)	119/1713 (6,9%)	132/4777 (2,8%)	<0,001
Tirofiban	20/1449 (1,4%)	82/1615 (5,1%)	173/1713 (10,1%)	275/4777 (5,8%)	<0,001
Inibidor da ECA	890/1449 (61,4%)	1059/1615 (65,6%)	1263/1713 (73,7%)	3212/4777 (67,2%)	<0,001
Estatina	1302/1449 (89,9%)	1467/1615 (90,8%)	1576/1713 (92%)	4345/4777 (91%)	0,108
Lovastatina	0/1293 (0%)	0/1461 (0%)	1/1568 (0,1%)	1/4322 (0%)	
Pravastatina	40/1293 (3,1%)	44/1461 (3%)	56/1568 (3,6%)	140/4322 (3,2%)	
Sinvastatina	581/1293 (44,9%)	619/1461 (42,4%)	914/1568 (58,3%)	2114/4322 (48,9%)	
Rosuvastatina	102/1293 (7,9%)	103/1461 (7%)	60/1568 (3,8%)	265/4322 (6,1%)	
Atorvastatina	570/1293 (44,1%)	695/1461 (47,6%)	537/1568 (34,2%)	1802/4322 (41,7%)	
Dupla Terapia antiplaquetária	1211/1449 (83,6%)	1463/1615 (90,6%)	1649/1713 (96,3%)	4323/4777 (90,5%)	<0,001
Terapia completa	787/1449 (54,3%)	1062/1615 (65,8%)	1116/1713 (65,1%)	2965/4777 (62,1%)	<0,001

*Valor de p: Teste Qui-quadrado. Dupla Terapia antiplaquetária: Aspirina e inibidor de P2Y12. Terapia completa: Dupla Terapia antiplaquetária, Anticoagulante Parenteral, Estatina e Betabloqueador.*

Dos 1.714 pacientes que apresentaram IAMcSST, alguma modalidade de reperfusão do miocárdio (fibrinólise ou intervenção coronariana percutânea primária) foi realizada em 1.412 (82,4%) indivíduos. Em análise da prescrição das terapias de reperfusão no IAM, observam-se percentuais distintos e decrescentes, de acordo com a região federativa brasileira: 87,3%, 84,5%, 72,8%, 66,7% e 65,7%, (p < 0,001), nas regiões sul, sudeste, nordeste, centro-oeste e norte do Brasil, respectivamente. Na medida da elevação da gravidade, na apresentação clínica desses três componentes das SCA, observou-se uma progressiva elevação da prescrição “estratégias invasivas”, seja de cinecoronariografia (68,0%, 83,1% e 90,4%; p < 0,001), bem como da efetiva realização de procedimento de revascularização do miocárdio (38,2%, 54,4% e 76,4%; p < 0,001), nos casos de angina instável, IAM SSST e IAM CSST, respectivamente. O procedimento de revascularização preferencial nesses pacientes foi a intervenção coronária percutânea com taxas superiores a 95% da utilização de *stents* coronários em pacientes tratados de forma percutânea. O percentual de revascularização percutânea dentre todos os pacientes com SCA variou conforme o diagnóstico: angina instável, IAM SSST e IAM CSST (33,6%, 47,4% e 75,1%, respectivamente; p <0,001).

Na alta hospitalar, observou-se que a prescrição de um inibidor da P2Y12 variou conforme o tipo de SCA (66,4% na angina instável, 77,7% no IAM SSST e 90,9% no IAM CSST; p<0,001), e o tipo de tratamento da doença coronária (ICP (94,2%), cirurgia (25%) ou clínico (66,2%); p <0,001).

**Figura 1:**
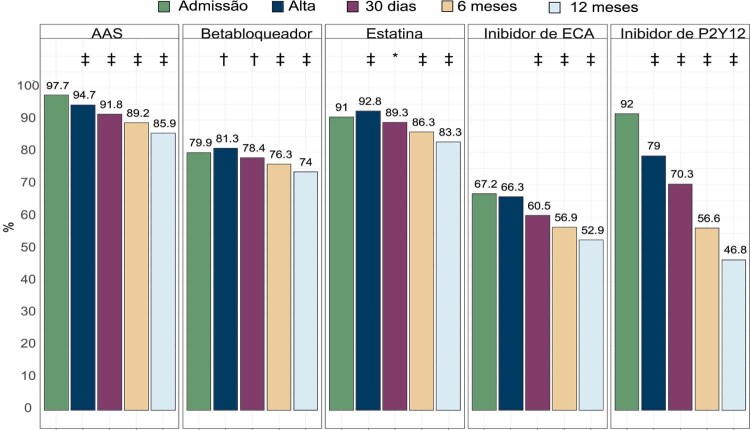
- Adesão a terapias baseadas em evidência no seguimento de 1 ano. Para a comparação da continuidade da prescrição do medicamento nos seguimentos com a admissão, foi ajustado um modelo de Equações de Estimação Generalizada (EEG) para dados binários, para considerar a dependência entre as observações. ‡ Valor de p < 0,001; Comparação entre o seguimento e a admissão; † Valor de p < 0,01; Comparação entre o seguimento e a admissão; * Valor de p < 0,05; Comparação entre o seguimento e a admissão.

A evolução das principais terapias da admissão para a alta hospitalar, ao final de 30 dias e em 6 e 12 meses demonstra redução progressiva no uso das terapias recomendadas, especialmente do uso de inibidores do receptor P2Y12 ( Figura 1 ).

### Desfechos clínicos

**Figura 2:**
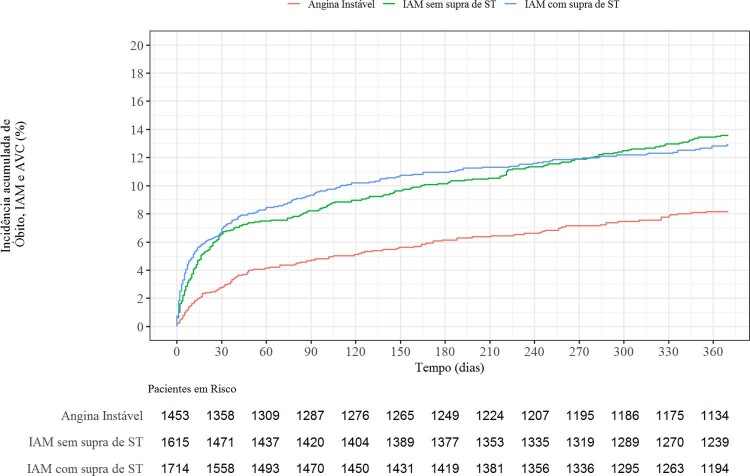
– *Desfechos clínicos em 1 ano de acordo com o diagnóstico.*

Os desfechos clínicos foram mensurados cumulativamente ao final dos 12 meses de evolução ( Figura 2 ). Dentre portadores de AI, não foi identificada uma associação entre ocorrência de eventos combinados (mortalidade, reinfarto ou AVE), ao final de 12 meses, e realização de procedimento de revascularização do miocárdio ( Tabela 3 ). Na evidência de IAM SSST, observou-se redução significativa da ocorrência de eventos cardiovasculares maiores, inclusive mortalidade cardiovascular, dentre aqueles submetidos ou não a um procedimento de revascularização do miocárdio, respectivamente (mortalidade = 6,29 por 100 pessoas ano versus 12,06 por 100 pessoas ano; p < 0,001 e desfechos cardiovasculares maiores = 13,18 por 100 pessoas ano versus 17,96 por 100 pacientes-ano; p = 0,038). Pacientes acometidos por IAM CSST apresentaram significativa redução das taxas de mortalidade e de ocorrência de eventos cardiovasculares maiores quando submetidos a revascularização do miocárdio (mortalidade = 8,02 por 100 pessoas ano versus 18,54 por 100 pessoas ano; p < 0,001 e eventos cardiovasculares = 13,11 por 100 pessoas anos versus 21,69 por 100 pessoas anos; p < 0,001). Em análise multivariada ( Tabela 4 ), os seguintes fatores estiveram associados à ocorrência de eventos cardiovasculares maiores: idade, atendimento público, IAM, AVE, insuficiência renal, diabetes e qualidade da terapia (completa ou não). Não houve interação significativa entre as covariáveis.

**Tabela 3:** – Relação entre procedimento de revascularização e desfecho clínico nos 3 tipos de síndrome coronária aguda

Eventos nos pacientes revascularizados em relação aos não-revascularizados	Angina Instável HR [95% IC]	IAM sem supra de ST HR [95% IC]	IAM com supra de ST HR [95% IC]
Sangramento Grave	2,03 [0,75 ; 5,44]	1,15 [0,55 ; 2,41]	1,28 [0,37 ; 4,50]
Parada Cardiorrespiratória	0,27 [0,09 ; 0,79]	0,54 [0,34 ; 0,87]	0,54 [0,36 ; 0,83]
Reinfarto do miocárdio	1,69 [1,03 ; 2,76]	1,28 [0,85 ; 1,90]	0,87 [0,53 ; 1,43]
Acidente vascular encefálico (AVE)	1,18 [0,26 ; 5,28]	0,80 [0,30 ; 2,13]	1,02 [0,34 ; 3,11]
Óbito	0,33 [0,17 ; 0,65]	0,53 [0,37 ; 0,76]	0,45 [0,33 ; 0,63]
Óbito Cardiovascular	0,45 [0,20 ; 1,06]	0,43 [0,28 ; 0,66]	0,43 [0,31 ; 0,62]
Desfecho Combinado	0,97 [0,66 ; 1,42]	0,75 [0,57 ; 0,98]	0,64 [0,48 ; 0,85]

*Desfecho Combinado: Óbito, Reinfarto do miocárdio e AVE. HR: Hazard Ratio*

**Tabela 4:** – Análise multivariada de fatores associados à ocorrência de eventos combinados (AVE, reinfarto ou óbito)

	Multivariada	

Variáveis	HR [95% IC ]	Valor de p
Idade		
Idade (aumento de 5 anos)	1,16 [1,11;1,20]	<0,001
Sexo		
Feminino	1,10 [0,91;1,33]	0,328
Atendimento (Saúde Suplementar/Particular)		
Saúde Suplementar/Particular	0,57 [0,47;0,69]	<0,001
Dislipidemia		
Sim	0,98 [0,81;1,19]	0,826
IAM		
Sim	1,29 [1,03;1,63]	0,030
Angina		
Sim	0,95 [0,78;1,16]	0,613
Hipertensão		
Sim	1,08 [0,85;1,36]	0,534
AVE		
Sim	1,38 [1,06;1,80]	0,017
Insuficiência renal		
Sim	2,08 [1,59;2,71]	<0,001
Diabetes		
Sim	1,48 [1,23;1,78]	<0,001
ICC		
Sim	1,10 [0,83;1,45]	0,502
Intervenção coronária percutânea		
Sim	1,00 [0,80;1,27]	0,961
Cirurgia de RM		
Sim	0,94 [0,72;1,25]	0,684
Uso de AAS		
Sim	1,18 [0,96;1,47]	0,120
Tabagismo		
Nunca	ref	ref
Ex-tabagista	1,22 [0,99;1,50]	0,055
Atual	1,27 [1,00;1,62]	0,047
Terapia completa		
Sim	0,72 [0,61;0,86]	<0,001
Diagnóstico Final		
Angina Instável	ref	ref
IAM sem supra de ST	1,76 [1,39;2,23]	<0,001
IAM com supra de ST	2,04 [1,59;2,62]	<0,001

*Variáveis com valor de p < 0,15 em análise “univariada” foram candidatas para a análise multivariada, ** As variáveis que apresentaram valor de p > 0,15 na análise univariada foram: Transferência de outro serviço, História familiar de doença coronariana, Obesidade Abdominal, Sedentarismo e Doença arterial periférica.

A taxa de eventos nos usuários do SUS foi de 16,6 por 100 pacientes-ano, ao passo que, na rede privada/convênio, foi de 9,10 por 100 pacientes-ano (p<0,01). Na análise por região, a taxa de óbito em 1 ano foi significativamente maior na região norte (19,8%; IC95% 12,6-27,0), seguida pelas regiões sudeste (8,0%; IC 95% 7,0-9,1), sul (6,8%; IC 95% 4,8-8,7) e nordeste (5,6%; IC 95% 3,7-7,5). A região centro-oeste teve menor representação de pacientes com taxa de mortalidade intermediária entre o norte e o restante do país (14,2%; IC95% 2,8-25,5). Na comparação dos preditores de eventos entre a região norte e as 3 regiões com taxa de eventos menores (sul, sudeste e nordeste), houve maior frequência de IAM cSST (51,0% x 35,3%; p <0,01), atendimento pelo SUS (100% x 51,8%; p < 0,01) e terapia incompleta nos pacientes incluídos na região norte do país (47,9% x 37,2%; p < 0,01).

## Discussão

Neste, que é o maior registro prospectivo brasileiro de pacientes com SCA, observou-se que mais de dois terços dos eventos são classificados como infarto agudo do miocárdio na admissão. O perfil dos pacientes indica predomínio do sexo masculino (70%), quase um terço de diabéticos e a hipertensão arterial sistêmica como o fator de risco mais comum (74,6%). Quase 40% dos pacientes não recebeu pelo menos uma das terapias baseadas em evidência na internação e a conformidade às recomendações variou de acordo com a região, com o tipo de SCA e com a estratégia de revascularização. O risco de eventos cardiovasculares maiores em 1 ano foi de 13,6 por 100 pessoas ano e dos sete fatores associados a estes eventos, dois são relacionados a aspectos do atendimento: financiamento (público x privado) e qualidade da terapia (completa ou não).

A publicação com resultados parciais do ACCEPT feita em 2013^10^ tinha incluído 2.584 pacientes e analisado 2.485, após exclusão de casos não confirmados de SCA. Na presente análise, foram acrescidos 2.463 pacientes, o que totalizou 5.047 arrolados ao final do estudo (4.782 casos de SCA confirmados). Além do tamanho amostral, uma outra diferença marcante é o tempo de seguimento, pois, assim como a publicação de dados intermediários do ACCEPT,^10^ a maioria das publicações de registros nacionais em SCA reportaram dados apenas de desfechos intra-hospitalares ou de seguimento de 30 dias.^12 , 13^ O estudo ERICO, publicado em 2015, reportou o seguimento de 1 ano dos pacientes internados por SCA em um hospital público de São Paulo/SP.^14^ Dessa forma, a presente análise incluiu, de forma inédita, dados do seguimento de 12 meses de uma grande população contemporânea de pacientes com SCA de diversas regiões do Brasil, incluindo a análise da conformidade da prescrição médica às recomendações de terapias baseadas em evidência em 12 meses. A adesão inicial da prescrição médica identificada no ACCEPT foi semelhante ao que foi encontrado em registros de países em desenvolvimento,^15^ embora tenha se apresentado abaixo do encontrado em centros que participam de programa de qualidade nos mesmos países.^9^ No seguimento de 1 ano, houve queda na prescrição de todas as terapias, especialmente inibidores de P2Y12, cuja utilização estava bem abaixo daquela identificada em registros internacionais de países desenvolvidos.^16 , 17^

No seguimento de 12 meses, identificou-se também um risco residual de 13,6 por 100 pacientes-ano para eventos cardiovasculares maiores (reinfarto, morte, AVE). A relação destes eventos com a realização de revascularização pareceu mais clara nos casos de IAM, pois na angina instável a análise combinada dos desfechos CV não mostrou menor taxa nos pacientes submetidos a revascularização. Por ser uma análise observacional (não randomizada), tal evidência não permite estabelecer relação de causa-efeito, mas reforça a validade externa do conceito gerado por ensaios clínicos sobre o benefício de revascularização em pacientes com SCA, especialmente aqueles de maior risco.^18 , 19^

Uma forma de minimizar o viés de uma análise observacional é incluir as diversas variáveis coletadas em um modelo que permita identificar a relação individual de forma independente. Dentre os fatores identificados em análise multivariada, dois foram relacionados ao atendimento: público x privado e qualidade da terapia (completa ou não). A qualidade da terapia se baseou nas recomendações baseadas em evidência para essa população.^6 , 7^ A relação de desfecho com qualidade da terapia já foi demonstrada em diversas publicações prévias^8 , 15^ e apresenta importância adicional para a validade externa dos efeitos identificados em ensaios clínicos controlados. A explicação para a diferença de desfechos identificada entre os pacientes do atendimento público ou privado, poderia ser resultante da diferença na qualidade assistencial. Entretanto, como o modelo multivariado identificou que o atendimento privado apresenta associação com melhores desfechos de forma independente da qualidade da terapia, uma possível explicação seria a própria classe social/educacional dos pacientes. Esta informação não foi coletada para inclusão direta no modelo multivariado da presente análise, mas, em estudos prévios, elas foram identificadas como fatores associados a desfechos clínicos nesta população.^15 , 20^

### Limitações do estudo

Uma limitação do estudo seria referente ao perfil de pacientes, pois trata-se de um registro voluntário, cujos serviços participantes apresentaram capacidade de pesquisa clínica e, por consequência, os resultados podem não ser aplicáveis a populações que não se enquadrem nestas características (ex: hospitais com estrutura mais limitada). De qualquer maneira, mesmo em centros de maior potencial de qualidade assistencial, foram identificadas lacunas relevantes na aplicação da evidência científica. Outra limitação seria a avaliação da adesão às terapias baseadas em evidência, pois essa análise se baseou na adesão do médico em termos de prescrição de terapias baseadas em evidência. Não foram coletados dados sobre a elegibilidade, a real utilização das terapias prescritas e os motivos para a descontinuação da prescrição. Dessa maneira, tendo em vista que a não adesão por parte dos pacientes não foi avaliada no presente registro, a lacuna sobre o uso de terapias baseadas em evidência poderia ser ainda maior do que aquela encontrada no registro ACCEPT, o qual avaliou a prescrição médica. Finalmente, a análise de desfecho clínico apresenta limitações relacionadas à ausência de adjudicação de eventos e dados faltantes no seguimento de 12 meses de 410 pacientes. Não obstante, a avaliação de desfecho clínico em estudos observacionais pragmáticos habitualmente é realizada por notificação do médico investigador, sem a utilização de um comitê específico para adjudicação, o que representaria um cenário mais próximo da forma de identificação de eventos que ocorre na prática clínica real. Quanto ao seguimento, tendo em vista que as perdas de acompanhamento ocorreram em momentos distintos, as análises foram realizadas por modelo de Cox e, por consequência, os pacientes foram censurados no último contato registrado como forma de minimizar as variações na duração de seguimento.

## Conclusão

No maior registro prospectivo já publicado de pacientes com SCA no Brasil, identificou-se uma taxa média de eventos cardiovasculares maiores em 1 ano acima de 13 por 100 pacientes-ano, mas que atingiu mais de 16,6 por 100 pacientes-ano, no ambiente de atendimento público (SUS). Como há falhas na prescrição de terapias baseadas em evidência desde a admissão, e que se intensificam durante o seguimento, a elaboração de estratégias para aumentar a adesão da prescrição baseada em evidência poderia minimizar o risco de tais eventos na população brasileira.
