# Supplementary material for: Avaliação do Seguimento de um Ano dos Pacientes Incluídos no Registro Brasileiro de Síndromes Coronárias Agudas (ACCEPT)
Source: Arq Bras Cardiol. 2020 Jun 29;114(6):995–1003. [Article in Portuguese] doi: 10.36660/abc.20190879 (PMC8416119; doi:10.36660/abc.20190879)
Supplement: Translation

# Translation: One year follow-up Assessment of Patients Included in the Brazilian Registry of Acute Coronary Syndromes (ACCEPT)

**Authors:** Pedro Gabriel Melo de Barros e Silva, Otavio Berwanger, Elizabete Silva dos Santos, Antônio Carlos Sobral Sousa, Margaret Assad Cavalcante, Pedro Beraldo de Andrade, Fernando Carvalho Neuenschwander, Hugo Vargas, Jorge Ilha Guimarães, Jadelson Andrade, Angelo Amato Vincenzo de Paola, Marcus Vinicius Bolivar Malachias, Luiz Alberto Piva e Mattos, Dalton Bertolim Precoma, Fernando Bacal, Oscar Pereira Dutra

**Affiliations:** 1 Brazil Hospital do Coração - Pesquisa Clínica, São Paulo, SP - Brazil; 2 Brazil Hospital Samaritano Paulista – Cardiologia, São Paulo, SP - Brazil; 3 Brazil Hospital Israelita Albert Einstein, São Paulo, SP - Brazil; 4 Brazil Instituto Dante Pazzanese de Cardiologia, São Paulo, SP - Brazil; 5 Brazil PPGCS e HU da Universidade Federal de Sergipe (UFS), Aracaju, SE - Brazil; 6 Brazil Hospital São Lucas - Rede D`Or, Aracaju, SE - Brazil; 7 Brazil Universidade do Oeste Paulista (Unoeste), Presidente Prudente, SP - Brazil; 8 Brazil Hospital Regional de Presidente Prudente, Presidente Prudente, SP - Brazil; 9 Brazil Faculdade de Medicina de Marilia, Marilia, SP - Brazil; 10 Brazil Santa Casa de Marília, Marília, SP - Brazil; 11 Brazil Hospital Vera Cruz, Belo Horizonte, MG - Brazil; 12 Brazil Hospital São Vicente de Paulo, Passo Fundo, RS - Brazil; 13 Brazil Sociedade Brasileira de Cardiologia, Rio de Janeiro, RJ - Brazil; 14 Brazil Hospital da Bahia, Salvador, BA - Brazil; 15 Brazil Universidade Federal de São Paulo Escola Paulista de Medicina, São Paulo, SP - Brazil; 16 Brazil Faculdade de Ciências Médicas de Minas Gerais, Belo Horizonte, MG - Brazil; 17 Brazil Instituto de Hipertensão Arterial - Diretoria Clínica, Belo Horizonte, MG - Brazil; 18 Brazil Rede D`Or, São Paulo, SP – Brazil; 19 Brazil Rede D`Or, Recife, PE - Brazil; 20 Brazil Pontifícia Universidade Católica do Paraná - Escola de Medicina, Curitiba, PR – Brazil; 21 Brazil Sociedade Hospitalar Angelina Caron – Cardiologia, Campina Grande do Sul, PR - Brazil; 22 Brazil Universidade de São Paulo Faculdade de Medicina Hospital das Clínicas Instituto do Coração, São Paulo, SP - Brazil; 23 Brazil Instituto de Cardiologia – Fundação Universitária de Cardiologia do Rio Grande do Sul, Porto Alegre, RS – Brazil

**Keywords:** Acute Coronary Syndrome, Myocardial Infarction, Risk factors, Medical Records/ statistics& numeral data, Multicenter Studies

## Abstract

**Background:**

There is lack of prospective data on evolution within one year of acute coronary syndromes (ACS) in a representative population of Brazilian patients.

**Objectives:**

To assess the prescription of evidence-based therapies, the incidence of severe outcomes and the predictors for these outcomes in a multicenter Brazilian registry of ACS patients.

**Methods:**

The ACCEPT is a prospective observational study, which included patients hospitalized with a diagnostic of ACS in 47 Brazilian hospitals. The patients were followed for a 1 year and data were collected on the medical prescription and the occurrence of major cardiovascular events (cardiovascular mortality, reinfarction and cerebrovascular accident - CVA). Values of p < 0.05 were considered statistically significant.

**Results:**

A total of 5,047 patients were included in this registry from August 2010 to April 2014. The diagnosis of ACS was confirmed in 4,782 patients (94.7%) and, among those, the most frequent diagnosis was ACS with ST segment elevation (35.8%). The rate of major cardiovascular events was 13.6 % within 1 year. Adherence to prescription of evidence-based therapy at admission was of 62.1%. Age, public service, acute myocardial infarction, CVA, renal failure, diabetes and quality of therapy were associated independently with the occurrence of major cardiovascular events.

**Conclusions:**

During the one-year follow-up of the ACCEPT registry, more than 10% of the patients had major cardiovascular events and this rate ranged according with the quality of therapy. Strategies must be elaborated to improve the use of evidence-based therapies to minimize the cardiovascular events among the Brazilian population. (Arq Bras Cardiol. 2020; 114(6):995-1003)

## Introduction

The group of cardiovascular diseases, particularly acute coronary syndrome (ACS), represents the leading cause of mortality and disability in Brazil and worldwide.^1 - 3^ In addition to its current high frequency, there is a perspective of increase in this group of diseases in developing countries, such as Brazil.^1 - 5^ Despite the high morbidity and mortality of ACS currently, several strategies of proven efficacy to reduce the risk of complications in these patients have been developed.^6 , 7^ However, there are flaws in evidence-based therapies when applied to ACS patients, as has been identified in previous clinical practice registries.^8 - 10^ Those multicenter registries assessed mainly the intra-hospital period or a 30-day period from the acute event. However, they lacked long-term data on the follow-up of these patients.^8 - 10^ Among the previous 30-day follow-up database is the partial data release (without complete sample data) of the ACCEPT study.^10^ As previously reported in the 30-day follow-up partial release,^10^ the ACCEPT study group intended to continue the investigation, with the enrollment of a greater number of patients and the inclusion of 12-month follow-up data. Thus, the present analysis performed, once more, the assessment of the baseline characteristics and initial adherence of medical prescriptions to evidence-based therapies in a larger population (about twice as many patients compared to the initial publication with the intermediate data) and included data on the incidence rate of severe clinical outcomes during the follow-up.

## Objectives

In addition to the final results after 30 days with the overall study population, this one year follow-up assessment has the following objectives:

- To assess the rate of major cardiovascular events within 12 months in a sample of Brazilian post-ACS patients;

- To evaluate the conformity of medical prescriptions to evidence-based therapies within 12 months in a sample of Brazilian post-ACS patients;

- To identify predictors of major cardiovascular events within 12 months in a sample of Brazilian post-ACS patients.

## Methods

### Study Design

The ACCEPT (Acute Coronary Care Evaluation of Practice Registry) registry is a project conceived by the Brazilian Society of Cardiology (Sociedade Brasileira de Cardiologia - SBC), whose methods have been previously published.^10 , 11^ In sum, it is a prospective, voluntary, multicenter study, which gathered 53 centers from the five Brazilian federal regions, with the following distribution: Southeast (50.9%), Northeast (13.2%), South (24.5%), Midwest (5.7%) and North (5.7%). Patient inclusion occurred from August 2010 to April 2014, in public hospital care centers (Unified Health System - SUS), health maintenance organizations, or private health care, according with the following distribution: SUS 2669/4782 (55.8%), health maintenance organizations 1968/4782 (41.2%) and private hospitals 145/4782 (3%).

### Study participants

Patients diagnosed with the different types of ACS were included: unstable angina (UA), acute myocardial infarction (MI) without ST-segment elevation (NSTEMI) and with ST-segment elevation (STEMI). The main inclusion criteria were: ischemic symptoms of suspected ACS associated with ischemia-like ECG changes and/or myocardial injury biomarkers above the upper limit of normality. Patients transferred from other institutions with more than 12 hours after symptoms onset were excluded.

### Study procedures and variables analyzed

The study procedures and variables analyzed in the ACCEPT study have been previously published.^10 , 11^ In sum, data collection occurred at admission (index visit) and a second data collection was performed after 7 days or at discharge (whichever occurred first). After these two first visits, the study included visits at 30 days, 6 months and 12 months, which could take place in person, at routine medical care, or by phone.

Due to the pragmatic features of the study, the identification of patients’ comorbidities (e.g.: arterial hypertension, dyslipidemia) could be performed as follows: patients’ self-assessment, use of medication (antihypertensive and lipid-lowering) or investigators’ evaluation (in the latter case, the centers were oriented to follow the recommendations on diagnosis criteria adopted by the current guidelines of the Brazilian Society of Cardiology). Physical examination data could be obtained by direct measurement (obesity was defined by BMI > 30 Kg/m^2^ ). Other criteria were based on the registry of medical records of a variable collected by interview (e.g.: stress, ex-smoker if cessation date was > 6 months).

The evidence-based treatment plan that was considered in the ACCEPT was not modified throughout the study and was based on current guidelines.^6 , 7^ This treatment plan can be divided as follows:

- Index event admission: Double antiaggregation, parenteral anticoagulant, statin and betablocker in addition to reperfusion therapy in case of STEMI.

- Outpatient therapy post discharge: Double antiaggregation, statin, beta-blocker and ACE inhibitors/ARBs.

The cardiovascular events of interest analyzed in the population included were: cardiovascular mortality, non-fatal cardiac arrest, reinfarction and cerebrovascular accident (CVA).^10 , 11^ These outcomes were reported by the investigator according to recommended criteria,^10 , 11^ without an independent adjudication committee to confirm the events.

### Statistical analysis

The analysis of normally distributed continuous variables was performed using histograms. Normally distributed continuous variables were described as mean ± standard deviation. The means were compared between the three diagnosis groups using the variance analysis ANOVA. Categorical variables were described by absolute and relative frequencies. The proportions were compared using the Chi-square test or Fisher’s exact test. Generalized Estimating Equations (GEE) models were used to assess drug therapy over time. To compare the major cardiovascular events according with the final diagnosis, Cox proportional hazards model and Kaplan-Meier curves were used. The identification of independent predictors for the composite endpoint (cerebrovascular accident (CVA), reinfarction and death) was performed using Cox proportional hazards model with the final diagnosis and the baseline factor analyzed. This analysis of predictors was initially performed in a univariate fashion and variables with a p <0.15 were included in the multivariate analysis. P-values were presented as two-sided and p < 0.05 was considered statistically significant in the final analyses. Additionally, in the multivariate analysis, an interaction test was performed between the selected variables. All analyses were performed using R Statistical software, version 3.6.1.

## Results

Between August 2010 and April 2014, 5,047 patients were recruited from this nationwide registry, 265 of whom (5.25%) had undiagnosed chest pain and were excluded from the clinical follow-up because they did not fulfill the research inclusion criteria. Thus, 4,782 ACS patients were actually included in the analysis and followed in this prospective registry, in 53 hospitals from the 5 Brazilian federal regions. In a total of 410 patients (8.6%), it was not possible to obtain the final 12-month data.

### Baseline Characteristics

The patients’ clinical profile revealed the inclusion of approximately 70% of patients diagnosed with AMI at admission, almost one-third had diabetes, and around 90% presented at least one risk factor, with the most frequent being systemic arterial hypertension ( Table 1 ).

**Table 1:** – Baseline characteristics of the patients included according to the type of acute coronary syndrome

Patients’ final diagnosis					

	Unstable Angina (n=1453)	AMI without ST elevation (n=1615)	AMI with ST elevation (n=1714)	Total (n=4782)	p-value
Age; mean ± SD	63.9 ± 11.9 (n=1449)	64.7 ± 12.4 (n=1603)	60.8 ± 12.4 (n=1702)	63.1 ± 12.4 (n=4754)	<0.001^(1)^
Sex (Female)	588/1453 (40.5%)	489/1615 (30.3%)	460/1714 (26.8%)	1537/4782 (32.1%)	<0.001
Transferred from another service (Yes)	179/1451 (12.3%)	393/1614 (24.3%)	803/1713 (46.9%)	1375/4778 (28.8%)	<0.001
Healthcare (Supplemental Insurance/Private)	757/1453 (52.1%)	775/1615 (48%)	581/1714 (33.9%)	2113/4782 (44.2%)	<0.001
Systolic Arterial Pressure; mean ± SD	138.1 ± 24.1 (n=1452)	137.9 ± 28 (n=1615)	131.5 ± 26 (n=1713)	135.7 ± 26.4 (n=4780)	<0.001^(1)^
Diastolic Arterial Pressure; mean ± SD	81.4 ± 13.9 (n=1452)	81.3 ± 16.4 (n=1615)	80.4 ± 16.4 (n=1713)	81 ± 15.7 (n=4780)	0.142^(1)^
Heart rate; mean ± SD	74.6 ± 15.3 (n=1452)	77.6 ± 18 (n=1615)	79.4 ± 17.2 (n=1713)	77.4 ± 17 (n=4780)	<0.001^(1)^
Dyslipidemia	971/1453 (66.8%)	915/1615 (56.7%)	734/1713 (42.8%)	2620/4781 (54.8%)	<0.001
Previous AMI	507/1451 (34.9%)	535/1614 (33.1%)	267/1713 (15.6%)	1309/4778 (27.4%)	<0.001
Angina history	774/1452 (53.3%)	554/1614 (34.3%)	406/1713 (23.7%)	1734/4779 (36.3%)	<0.001
Hypertension	1197/1453 (82.4%)	1252/1615 (77.5%)	1116/1713 (65.1%)	3565/4781 (74.6%)	<0.001
Family History of Coronary Disease	643/1453 (44.3%)	658/1615 (40.7%)	699/1713 (40.8%)	2000/4781 (41.8%)	0.081
CVA	137/1453 (9.4%)	125/1615 (7.7%)	98/1713 (5.7%)	360/4781 (7.5%)	<0.001
Stress and/or Depression	506/1451 (34.9%)	419/1614 (26%)	466/1713 (27.2%)	1391/4778 (29.1%)	<0.001
Renal Failure	88/1452 (6.1%)	99/1615 (6.1%)	72/1713 (4.2%)	259/4780 (5.4%)	0.021
Diabetes Mellitus	477/1453 (32.8%)	582/1615 (36%)	453/1713 (26.4%)	1512/4781 (31.6%)	<0.001
Diabetes treated with insulin	134/474 (28.3%)	150/582 (25.8%)	84/453 (18.5%)	368/1509 (24.4%)	-
Heart Failure	180/1452 (12.4%)	156/1615 (9.7%)	87/1713 (5.1%)	423/4780 (8.8%)	<0.001
Percutaneous Coronary Intervention	489/1450 (33.7%)	406/1614 (25.2%)	209/1713 (12.2%)	1104/4777 (23.1%)	<0.001
CABG	223/1452 (15.4%)	213/1615 (13.2%)	68/1713 (4%)	504/4780 (10.5%)	<0.001
Previous use of ASA	861/1453 (59.3%)	703/1615 (43.5%)	383/1713 (22.4%)	1947/4781 (40.7%)	<0.001
Abdominal Obesity	531/1452 (36.6%)	552/1615 (34.2%)	521/1713 (30.4%)	1604/4780 (33.6%)	0.001
Sedentary lifestyle	949/1453 (65.3%)	968/1615 (59.9%)	962/1713 (56.2%)	2879/4781 (60.2%)	<0.001
Peripheral arterial disease	130/1453 (8.9%)	135/1615 (8.4%)	126/1713 (7.4%)	391/4781 (8.2%)	0.252
Smoking					
Never	761/1453 (52.4%)	756/1615 (46.8%)	664/1713 (38.8%)	2181/4781 (45.6%)	<0.001
Ex-smoker	487/1453 (33.5%)	503/1615 (31.1%)	387/1713 (22.6%)	1377/4781 (28.8%)	
Current smoker	205/1453 (14.1%)	356/1615 (22%)	662/1713 (38.6%)	1223/4781 (25.6%)	

*P-value: Chi-square test. (1) ANOVA test*

### Medical prescription adherence to evidence-based therapies

The prescription adopted soon after admission shows that full adherence to medications currently recommended in the guidelines was of 62.1 % ( Table 2 ). This adherence includes dual antiplatelet therapy (aspirin/P2Y12 inhibitor) combined with parenteral anticoagulants, statins and betablockers.

**Table 2:** – Use of medication by patients with Acute Coronary Syndrome at the admission stage

Medication	Unstable Angina	AMI without ST elevation	AMI with ST elevation	Total	p
ASA	1399/1449 (96.5%)	1580/1615 (97.8%)	1688/1713 (98.5%)	4667/4777 (97.7%)	0.001
Betablocker	1144/1449 (79%)	1323/1615 (81.9%)	1352/1713 (78.9%)	3819/4777 (79.9%)	0.052
P2Y12 inhibitor	1239/1449 (85.5%)	1483/1615 (91.8%)	1671/1713 (97.5%)	4393/4777 (92%)	<0.001
Clopidogrel	1213/1449 (83.7%)	1401/1615 (86.7%)	1531/1713 (89.4%)	4145/4777 (86.8%)	<0.001
Prasugrel	11/1449 (0.8%)	17/1615 (1.1%)	15/1713 (0.9%)	43/4777 (0.9%)	0.685
Ticagrelor	23/1449 (1.6%)	80/1615 (5%)	149/1713 (8.7%)	252/4777 (5.3%)	<0.001
Parenteral Anticoagulant	1151/1449 (79.4%)	1468/1615 (90.9%)	1500/1713 (87.6%)	4119/4777 (86.2%)	<0.001
Enoxaparina	837/1449 (57.8%)	1039/1615 (64.3%)	1086/1713 (63.4%)	2962/4777 (62%)	<0.001
Fondaparinux	113/1449 (7.8%)	206/1615 (12.8%)	174/1713 (10.2%)	493/4777 (10.3%)	<0.001
Unfractionated heparin	214/1449 (14.8%)	240/1615 (14.9%)	282/1713 (16.5%)	736/4777 (15.4%)	0.319
GP IIb/IIIa Inhibitors	23/1449 (1.6%)	91/1615 (5.6%)	292/1713 (17%)	406/4777 (8.5%)	<0.001
Abciximab	3/1449 (0.2%)	10/1615 (0.6%)	119/1713 (6.9%)	132/4777 (2.8%)	<0.001
Tirofiban	20/1449 (1.4%)	82/1615 (5.1%)	173/1713 (10.1%)	275/4777 (5.8%)	<0.001
ACE inhibitor	890/1449 (61.4%)	1059/1615 (65.6%)	1263/1713 (73.7%)	3212/4777 (67.2%)	<0.001
Statin	1302/1449 (89.9%)	1467/1615 (90.8%)	1576/1713 (92%)	4345/4777 (91%)	0.108
Lovastatin	0/1293 (0%)	0/1461 (0%)	1/1568 (0.1%)	1/4322 (0%)	
Pravastatin	40/1293 (3.1%)	44/1461 (3%)	56/1568 (3.6%)	140/4322 (3.2%)	
Sinvastatin	581/1293 (44.9%)	619/1461 (42.4%)	914/1568 (58.3%)	2114/4322 (48.9%)	
Rosuvastatin	102/1293 (7.9%)	103/1461 (7%)	60/1568 (3.8%)	265/4322 (6.1%)	
Atorvastatin	570/1293 (44.1%)	695/1461 (47.6%)	537/1568 (34.2%)	1802/4322 (41.7%)	
Dual antiplatelet therapy	1211/1449 (83.6%)	1463/1615 (90.6%)	1649/1713 (96.3%)	4323/4777 (90.5%)	<0.001
Complete therapy	787/1449 (54.3%)	1062/1615 (65.8%)	1116/1713 (65.1%)	2965/4777 (62.1%)	<0.001

*P-value: Chi-square test. Dual antiplatelet therapy: Aspirin and P2Y12 inhibitor. Complete therapy: Dual antiplatelet therapy, Parenteral Anticoagulant, Statin and Betablocker.*

Out of the 1,714 patients presented with AMI (STEMI), 1,412 (82.4%) individuals were treated with some modality of reperfusion of the myocardium (either fibrinolysis or primary percutaneous coronary intervention). When analyzing the prescription of reperfusion therapies for AMI, there are distinct and decreasing percentages, according to the Brazilian federal region: 87.3%, 84.5%, 72.8%, 66.7% and 65.7%, (p < 0.001), for the South, Southeast, Northeastern, Midwest, and Northern Brazilian states, respectively. As the severity of the clinical presentation of these three components of the ACS increased, there was a progressive increase in the prescription of invasive strategies, either coronary angiography (68.0%, 83.1% and 90.4%; p < 0.001), or myocardial revascularization procedures (38.2%, 54.4% and 76.4%; p < 0.001), in case of unstable angina, NSTEMI and STEMI, respectively. The preferred revascularization procedure in these patients was percutaneous coronary intervention with rates > 95% of coronary stent use in patients treated percutaneously. The percentage of percutaneous revascularization among all ACS patients ranged according with the diagnosis: unstable angina, NSTEMI and STEMI (33.6%, 47.4% and 75.1%, respectively; p <0.001).

We observed that the prescription of a P2Y12 inhibitor at hospital discharge varied according with the type of ACS (66.4% for unstable angina, 77.7% for NSTEMI and 90.9% for STEMI; p<0.001), and type of coronary disease treatment the patient received (PCI (94.2%), surgical (25%) or clinical (66.2%); p <0.001).

The evolution of the main therapies, from admission to discharge, at the end of 30 days and in 6 and 12 months shows a progressive reduction in the use of the therapies recommended, especially in relation to therapy with the use of P2Y12 receptor inhibitors (Figure 1).

**Figure 1:** Adherence to evidence-based therapies in the first year of follow-up. To compare the continuity of medical prescription in follow-up with admission, a model of Generalized Estimating Equations (EEG) was adjusted for binary data, to take into consideration the dependence between observations. ‡ P-value < 0.001; Comparison between follow-up and admission; † P-value < 0.01; Comparison between follow-up and admission; * P value < 0.05; Comparison between follow-up and admission

### Clinical Outcomes

Clinical outcomes were measured cumulatively at the end of the first 12 months of evolution (Figure 2). Among patients with UA, there was no association between the occurrence of the composite events (mortality, reinfarction or cerebrovascular accident (CVA)) at the end of the first 12 months and the performance of myocardial revascularization procedure ( Table 3 ). In the presence of NSTEMI, a significant reduction was observed in the incidence of major cardiovascular events, including cardiovascular mortality, among those submitted or not to myocardial revascularization (mortality = 6.29 per 100 people/year *versus* 12.06 per 100 people/year; p < 0.001 and major cardiovascular outcomes = 13.18 per 100 people/year *versus* 17.96 per 100 patients/year; p = 0.038), respectively. STEMI patients had a significant reduction in mortality rates and incidence rates of major cardiovascular events when submitted to myocardial revascularization (mortality = 8.02 per 100 people/year *versus* 18.54 per 100 people/year; p < 0.001 and cardiovascular events = 13.11 per 100 people/year 21.69 per 100 people/year; p < 0.001). In the multivariate analysis ( Table 4 ), the following factors were associated with the occurrence of major cardiovascular events: age, public health care, AMI, CVA, renal failure, diabetes and quality of therapy (complete or not). There was no significant interaction between the covariables.

**Figure 2:** – *One-year clinical outcomes according with the diagnosis.*

**Table 3:** – Relationship between the revascularization procedure and clinical outcomes in the 3 types of acute coronary syndrome

Events in revascularized patients compared to non-revascularized patients	Unstable Angina HR [95% CI]	AMI without ST elevation HR [95% CI]	AMI with ST elevation HR [95% CI]
Severe Bleeding	2.03 [0.75 ; 5.44]	1.15 [0.55 ; 2.41]	1.28 [0.37 ; 4.50]
Cardiorespiratory Arrest	0.27 [0.09 ; 0.79]	0.54 [0.34 ; 0.87]	0.54 [0.36 ; 0.83]
Myocardial Reinfarction	1.69 [1.03 ; 2.76]	1.28 [0.85 ; 1.90]	0.87 [0.53 ; 1.43]
Cerebrovascular Accident (CVA)	1.18 [0.26 ; 5.28]	0.80 [0.30 ; 2.13]	1.02 [0.34 ; 3.11]
Death	0.33 [0.17 ; 0.65]	0.53 [0.37 ; 0.76]	0.45 [0.33 ; 0.63]
Cardiovascular death	0.45 [0.20 ; 1.06]	0.43 [0.28 ; 0.66]	0.43 [0.31 ; 0.62]
Composite endpoint	0.97 [0.66 ; 1.42]	0.75 [0.57 ; 0.98]	0.64 [0.48 ; 0.85]

*Composite endpoint: Death, Myocardial reinfarction and CVA. HR: Hazard Ratio.*

**Table 4:** – Multivariate analysis of factors associated with the occurrence of composite events (CVA, reinfarction or death).

	Multivariate	

Variables	HR [95% CI ]	p-value
Age		
Age (5-year increase)	1.16 [1.11;1.20]	<0.001
Sex		
Female	1.10 [0.91;1.33]	0.328
Healthcare (Supplemental Insurance/Private)		
Supplemental Insurance/Private	0.57 [0.47;0.69]	<0.001
Dyslipidemia		
Yes	0.98 [0.81;1.19]	0.826
AMI		
Yes	1.29 [1.03;1.63]	0.030
Angina		
Yes	0.95 [0.78;1.16]	0.613
Hypertension		
Yes	1.08 [0.85;1.36]	0.534
CVA		
Yes	1.38 [1.06;1.80]	0.017
Renal Failure		
Yes	2.08 [1.59;2.71]	<0.001
Diabetes		
Yes	1.48 [1.23;1.78]	<0.001
CHF		
Yes	1.10 [0.83;1.45]	0.502
Percutaneous coronary intervention		
Yes	1.00 [0.80;1.27]	0.961
CABG		
YES	0.94 [0.72;1.25]	0.684
ASA use		
Yes	1.18 [0.96;1.47]	0.120
Smoking		
Never	ref	ref
Ex-smoker	1.22 [0.99;1.50]	0.055
Current smoker	1.27 [1.00;1.62]	0.047
Complete therapy		
Yes	0.72 [0.61;0.86]	<0.001
Final Diagnosis		
Unstable Angina	ref	ref
AMI with ST elevation	1.76 [1.39;2.23]	<0.001
AMI with ST elevation	2.04 [1.59;2.62]	<0.001

*Variables with p values < 0.15 in the univariate analysis were included in the multivariable model. ** The variables with p values > 0.15 in the univariate analysis were: Transfer from another service, Family History of Coronary Disease, Abdominal Obesity, Sedentary Lifestyle and Peripheral Arterial Disease.

The rate of events among SUS patients was 16.6 per 100 patients/year, whereas in the private associated network it was 9.10 per 100 patients/year (p<0.01). In the analysis per federal region, the 1-year death rate was significantly higher in the Northern region (19.8%; CI95% 12.6-27.0), followed by the Southeast (8.0%; CI95% 7.0-9.1), South (6.8%; CI95% 4.8-8.7) and Northeast regions (5.6%; CI95% 3.7-7.5). The Midwest region had the lowest number of patients with intermediate mortality rate between the Northern region and the rest of Brazil (14.2%; CI95% 2.8-25.5). When comparing the predictors of events between the North region and the 3 regions with the lowest rates of events (South, Southeast and Northeast), we observed a greater incidence of STEMI (51.0% x 35.3%; p <0.01), SUS health care (100% x 51.8%; p < 0.01) and incomplete treatment among the patients from the North region of Brazil (47.9% x 37.2%; p < 0.01).

## Discussion

This database is considered the largest prospective Brazilian registry of ACS patients, and showed that more than two thirds of the events are classified as acute myocardial infarction at admission. The patients’ profile indicates a predominance of the male sex (70%), almost one-third of patients with diabetes, and systemic arterial hypertension as the most frequent risk factor (74.6%). Almost 40% of the patients did not receive at least one of the evidence-based therapies at admission and the conformity to recommendations varied according to the federal region, to the type of ACS and to the revascularization strategy adopted. The risk of major cardiovascular events within a year was 13.6 per 100 people/year and, out of the seven factors associated with these events, two are related with the health care characteristics: financing (public vs. private) and quality of therapy (complete or incomplete).

The ACCEPT partial results, released in 2013,^10^ included 2,584 patients and analyzed 2,485, after the exclusion of non-confirmed cases of ACS. In the present analysis, 2,463 patients were added, totaling 5,047 enrolled patients by the end of the study (4,782 cases of confirmed ACS). In addition to sample size, another marked difference is the follow-up length, because, similarly to the publication of the ACCEPT intermediate data,^10^ most publications of nationwide registries on ACS only reported data on intra-hospital outcomes or at 30 day of follow-up.^12 , 13^ The ERICO study, released in 2015, reported a one year follow-up of patients admitted due to ACS in a public hospital in the state of São Paulo.^14^ Thus, the present analysis included, in an unprecedented manner, 12-month follow-up data of a large contemporary population of ACS patients from several Brazilian federal regions, including the conformity assessment of medical prescriptions to the guidelines evidence-based therapies within 12 months. The initial adherence of medical prescriptions identified in the ACCEPT study was similar to the one seen in other developing countries,^15^ although it was below that of centers participating in quality programs in those same countries.^9^ During the one year follow-up, there was a decrease in prescription of all therapies, especially of P2Y12 inhibitors, whose administration was far below what was observed in the international registries of developed countries.^16 , 17^

At the 12-month follow-up, a residual risk of 13.6 per patient/year was also identified for major cardiovascular events (reinfarction, death and CVA). The connection of these events with the performance of revascularization seemed more evident in cases of AMI, because, in unstable angina, the combined analysis of cardiovascular outcomes did not reveal a lower rate among the patients submitted to revascularization. Since this is an observational non-randomized study, such evidence does not allow for the establishment of a cause-effect relation, but it reinforces the external validity of the concept generated by clinical trials on the benefits of revascularization for ACS patients, especially for those at a higher risk.^18 , 19^

One strategy to minimize the bias of observational studies is to include the several collected data in a model which allows to identify the individual relation in an independent manner. Among the factors identified in a multivariate analysis, two were related to health care: public *versus* private and quality of therapy (complete or not). The quality of therapy was based on the evidence-based recommendations for this population.^6 , 7^ The relation between outcome and quality of therapy has been demonstrated in several previous publications,^8 , 15^ and showed additional importance for the external validity of the effects identified in controlled clinical trials. The explanation for the difference in the outcomes identified between the patients from public or private-sector, could be owed to the difference in healthcare quality. However, since the multivariate model identified that private healthcare is associated with better outcomes independently of the quality of therapy, a possible explanation would be the patients’ social/educational level itself. These data were not collected for direct inclusion in the multivariate model of this analysis. However, in previous studies, they were identified as factors associated with clinical outcomes in this population.^15 , 20^

### Study limitations

One limitation of this study regards the patients’ profile, since this is a voluntary registry, whose participant services showed clinical research capacity. Therefore, the results may not be applicable to populations that do not fit these characteristics (for instance, hospitals with more limited structure). In any case, even in centers with potential for high-quality care, relevant gaps were identified when applying scientific evidence. Another limitation is related to assessment of adherence to evidence-based therapies, because this analysis was based on medical adherence in terms of the prescription of evidence-based therapies. We did not collect data on the eligibility, the actual administration of the therapies prescribed and the reasons for prescription discontinuity. Thus, considering that the adherence on the part of the patients was not assessed in this registry, the gap on the use of evidence-based therapies could be even bigger than that found in the ACCEPT registry, which evaluated the medical prescription. Finally, the clinical outcome assessment presents limitations regarding the absence of events adjudication and missing data of the 12-month follow-up of 410 patients. Nevertheless, the assessment of clinical outcomes in pragmatic observational studies is usually performed by notification of the investigator physician, without the use of a specific committee for adjudication, which would represent a scenario closer to the identification of events in real clinical practice. As for the follow-up, taking into account that the follow-up losses occurred at different moments, the analyses were performed using the Cox model. Consequently, the patients were censored in the last registered contact, in order to minimize the differences in follow-up length.

## Conclusion

In the largest prospective study ever published on patients with ACS in Brazil, we identified a mean rate of major cardiovascular events, within 1 year, above 13 per 100 patients/year, but which reached values above 16.6 per 100 patients/year in the public service context (SUS). Since there are flaws in the prescription of evidence-based therapies from admission, which are intensified during the follow-up, the creation of strategies to increase adherence of evidence-based prescription could minimize the risk of such events among the Brazilian population.
